# SARS-CoV-2 Omicron Replacement of Delta as Predominant Variant, Puerto Rico

**DOI:** 10.3201/eid2904.221700

**Published:** 2023-04

**Authors:** Gilberto A. Santiago, Hannah R. Volkman, Betzabel Flores, Glenda L. González, Keyla N. Charriez, Limari Cora Huertas, Steven M. Van Belleghem, Vanessa Rivera-Amill, Chelsea Major, Candimar Colon, Rafael Tosado, Laura E. Adams, Melissa Marzán, Lorena Hernández, Iris Cardona, Eduardo O’Neill, Gabriela Paz-Bailey, Riccardo Papa, Jorge L. Muñoz-Jordan

**Affiliations:** Centers for Disease Control and Prevention, San Juan, Puerto Rico (G.A. Santiago, H.R. Volkman, B. Flores, G.L. González, K.N. Charriez, C. Major, C. Colon, R. Tosado, L.E. Adams, G. Paz-Bailey, J.L. Muñoz-Jordan);; University of Puerto Rico Molecular Sciences and Research Center, San Juan (L.C. Huertas, S.M. Van Belleghem, R. Papa);; Ponce Research Institute, Ponce Health Sciences University, Ponce, Puerto Rico (V. Rivera-Amill);; Puerto Rico Department of Health, San Juan (M. Marzán, L. Hernández, I. Cardona);; Centers for Disease Control and Prevention, Atlanta, Georgia, USA (E. O’Neill)

**Keywords:** SARS-CoV-2, COVID-19, Omicron, Delta, coronavirus disease, severe acute respiratory syndrome coronavirus 2, viruses, respiratory infections, zoonoses, genomic surveillance, NGS, Puerto Rico

## Abstract

We reconstructed the SARS-CoV-2 epidemic caused by Omicron variant in Puerto Rico by sampling genomes collected during October 2021–May 2022. Our study revealed that Omicron BA.1 emerged and replaced Delta as the predominant variant in December 2021. Increased transmission rates and a dynamic landscape of Omicron sublineage infections followed.

Since the arrival of SARS-CoV-2 in Puerto Rico in March 2020, epidemic waves of COVID-19 have occurred on the island during the emergence of several variants of concern. Genomic surveillance conducted by partnered public health and academic groups reported an epidemic wave caused by the Alpha variant in April 2021, which coincided with the vaccination campaign for adults (*1*). Despite the detection of other variants of interest or concern, circulation of most of those variants was limited. According to the Puerto Rico Department of Health, the Delta variant has caused >49,000 confirmed cases since June 2021 (Appendix Figure 1). The epidemic wave began to decline in August 2021, reaching its lowest rate since the beginning of the Delta wave in December 2021. This decline was possibly associated with the successful COVID-19 vaccination program, in which 83% of the eligible population of Puerto Rico had received the initial series of COVID-19 vaccines by October 31, 2021 (*2*,*3*).

The first confirmed case of the Omicron variant in Puerto Rico was reported on November 29, 2021, and within a week, Omicron had replaced Delta to become the dominant circulating variant. The relatively low circulation of Delta, combined with Omicron’s high transmissibility and the waning of protective immunity before the vaccine booster campaign, might all have contributed to the rapid spread of this variant (*4*). The first Omicron peak was 9.1 times higher than any previous SARS-CoV-2 epidemic peak documented in Puerto Rico (Appendix Figure 1). By May 31, 2022, epidemic waves of the Omicron variant had caused ≈494,200 cases, peaking appreciably around January and May 2022.

We analyzed the Delta and Omicron sublineage turnover dynamics by using all the SARS-CoV-2 genomes from Puerto Rico sampled during October 2021–May 2022 available in GISAID (https://www.gisaid.org) as of June 8, 2022. By the end of December 2021, the Omicron sublineage BA.1 accounted for >60% of the sampled genomes, and the BA.1.1 sublineage dominated circulation until late March 2022 (Appendix Figure 2). Sublineage BA.2 began cocirculating with BA.1 sublineages in late January 2022 and remained at a low level until late March 2022, when it replaced BA.1.1 as the dominant sublineage. Subsequently, sublineage BA.2 caused another epidemic wave that peaked in mid-April 2022 (Appendix Figure 2). A period of sustained high transmission characterized the second Omicron wave; positive test rates for both antigen and molecular tests remained at >10% during April–August 2022.

To elucidate the emergence of the Omicron variant and subsequent replacement of Delta as the predominant variant, we used a phylogenetic approach to reconstruct the SARS-CoV-2 epidemic in Puerto Rico (Figure). We sequenced 2,377 SARS-CoV-2 complete genomes directly from reverse transcription PCR–positive diagnostic samples collected in Puerto Rico during the study period (*1*). We conducted time-calibrated phylogenetic analyses locally using the ncov augur/auspice pipeline (https://docs.nextstrain.org/projects/ncov/en/latest/index.html) with a custom subsample from the Puerto Rico dataset in GISAID and a custom subsample of contextual genomes derived from the GISAID NextRegion-North America dataset representative of the Americas, with an emphasis on the United States and the Caribbean region (Appendix). Our analysis demonstrates that the rapid emergence and expansion of the Omicron BA.1 sublineage during the decline of Delta is concordant with the epidemiologic trends reported during the same period (Figure; Appendix). Most genomes from Puerto Rico are closely related to genomes sampled in the United States, suggesting frequent virus introductions by infected travelers, as previously observed for other SARS-CoV-2 variants on the island (*1*). Tree topology shows the genomes from Puerto Rico grouping in multiple monophyletic clusters, suggesting that multiple importations propelled the expansion of subvariants. Our analysis also demonstrates the cumulative increase and subsequent expansion of BA.2 while it cocirculated with BA.1.1. We observed 2 distinct clusters of BA.2 genomes in the tree, suggesting 2 waves of BA.2 sublineage expansion. The first wave, detected in late March 2022, consisted of a variety of BA.2 sublineages, whereas the second wave in mid-May 2022 was caused by sublineage BA.2.12.1, which replaced all other sublineages (Figure).

**Figure:**
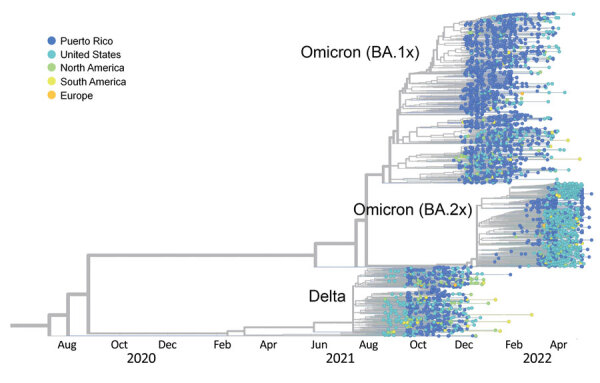
Decline of SARS-CoV-2 Delta variant and emergence of Omicron sublineages BA.1 and BA.2, illustrated by time-calibrated phylogenetic tree inferred with ncov augur/auspice workflow to represent the molecular evolution of the Delta variant in Puerto Rico since October 1, 2021, and subsequent expansion of the Omicron variant through May 30, 2022. Taxa labels are color-coded by geographic region of sampling to present the phylogenetic relatedness of viruses from Puerto Rico (dark blue) to viruses from the United States (light blue) and other regions of the world.

Our findings show that Omicron BA.1 seems to have emerged in a scenario favorable for rapid expansion, in which rates of transmission for Delta were low and protection from the vaccine or natural infection was waning in the population (*3*,*5*). An effective booster vaccination campaign by late 2021 could possibly have mitigated the BA.1 epidemic wave, although the occurrence of a second wave of BA.2 suggests this sublineage is more resistant to mRNA vaccines (*6*,*7*). The SARS-CoV-2 genomic epidemiology trends observed in Puerto Rico during the period of circulation of the Delta and Omicron variants resemble the trends reported in the United States (*8*). Additional increases in positive cases could be expected upon introduction of the BA.4 and BA.5 sublineages.

Appendix Additional information about SARS-CoV-2 Omicron replacement of Delta as predominant variant, Puerto Rico
