# Supplementary material for: SARS-CoV-2 Omicron Replacement of Delta as Predominant Variant, Puerto Rico
Source: Emerg Infect Dis. 2023 Apr;29(4):855–7. doi: 10.3201/eid2904.221700 (PMC10045710; doi:10.3201/eid2904.221700)
Supplement: Appendix — Additional information about SARS-CoV-2 Omicron replacement of Delta as predominant variant, Puerto Rico [file 22-1700-Techapp-s1.pdf]

Article DOI: https://doi.org/10.3201/eid2904.221700

EID cannot ensure accessibility for supplementary materials supplied by authors. Readers who have difficulty accessing supplementary content should contact the authors for assistance.

SARS-CoV-2 Omicron Replacement of Delta as Predominate Variant, Puerto Rico

Appendix

SARS-CoV-2 Genomes Used in this Study and Acknowledgment of Sequence Authors

All genome sequences and associated metadata in this dataset are published in GISAID's EpiCoV database, EPI_SET_220930nq. To view the contributors of each individual sequence with details such as accession number, virus name, collection date, originating lab and submitting lab, and the list of authors, please visit https://epicov.org/epi3/epi_set/220930nq for contextual genomes and list of genomes generated by this study.

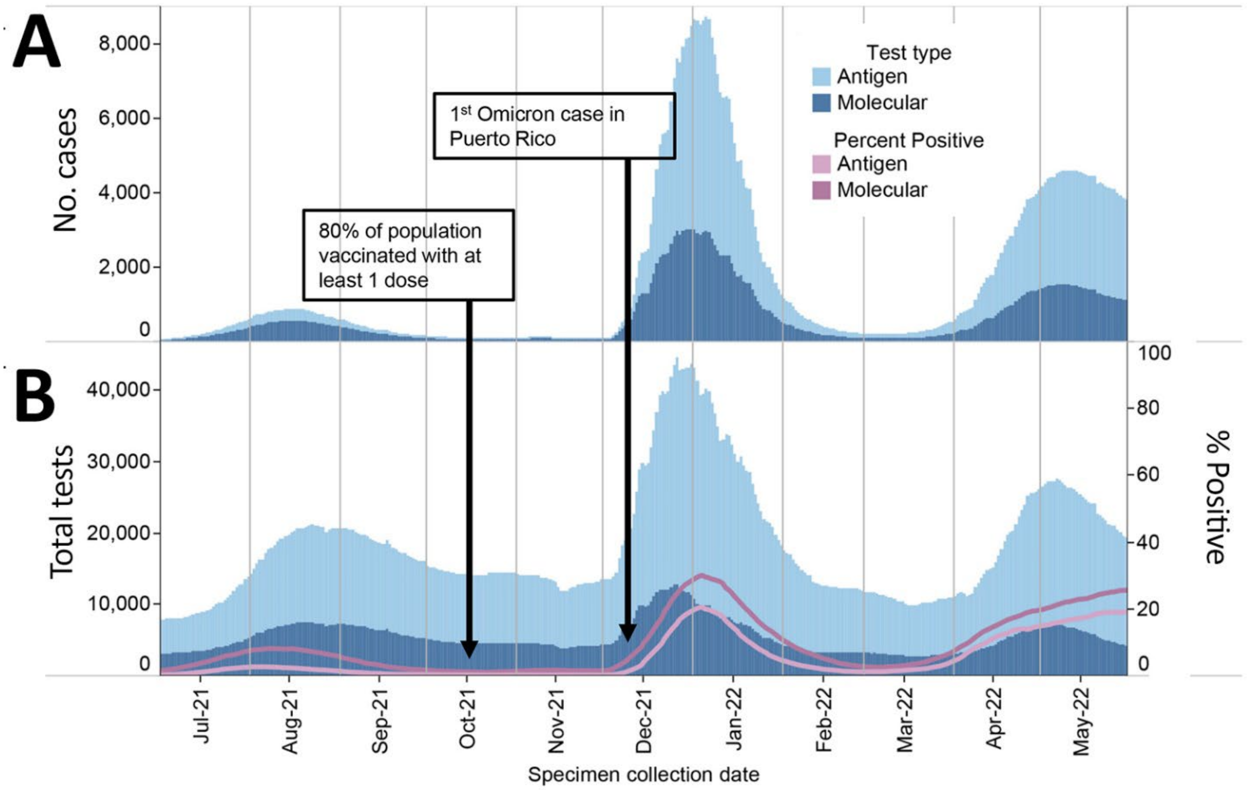

Appendix Figure 1. Epidemic curve of SARS-CoV-2 cases identified through molecular or antigen testing reported to the Puerto Rico Department of Health, July 2021-May 2022. A) Number of SARS-CoV-2 molecular or antigen tests reported to the Puerto Rico Department of Health and percent of reported tests positive, July 2021-May 2022. B) Epidemic curve, test counts, and percent of tests positive are visualized using a 21-day, centered moving average for clarity of trends.

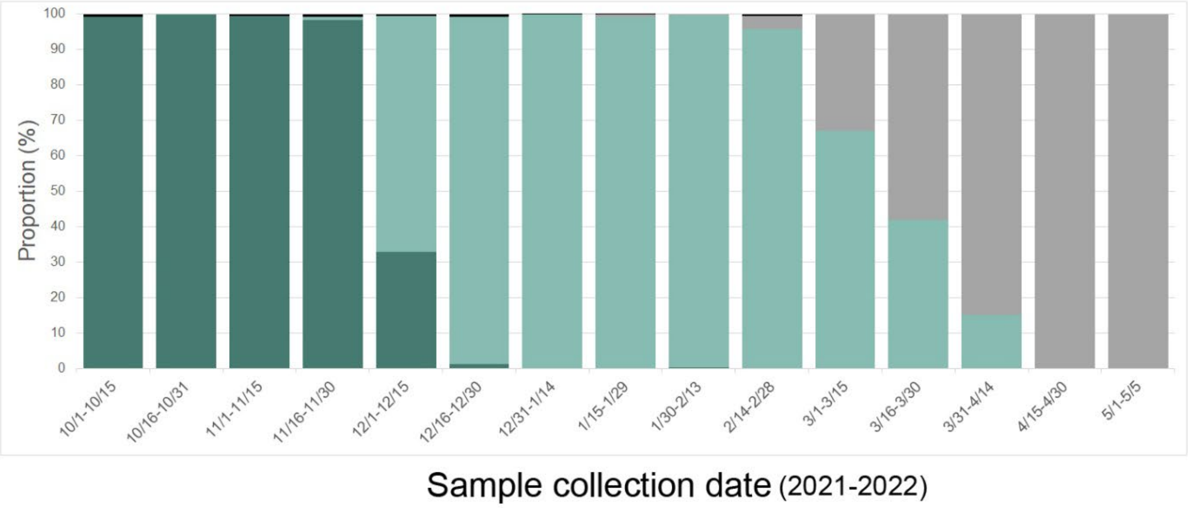

Appendix Figure 2. SARS-CoV-2 variant turn-over in Puerto Rico. Proportion of Delta variant and Omicron sub-lineages detected in Puerto Rico by genomic surveillance during October 1, 2021–May 30, 2022. All genomes sampled in Puerto Rico published in GISAID with collection dates within the study period were included (n = 4,204 genomes downloaded from GISAID on June 8, 2022). Delta variant is presented with bars in dark green, Omicron sublineage BA.1x in light green, and Omicron sublineage BA.2x in gray.
